# Metabolic imaging of *in vivo* myocardium

**DOI:** 10.1186/1532-429X-17-S1-P251

**Published:** 2015-02-03

**Authors:** Charles S Springer, Craig S Broberg, William D Rooney

**Affiliations:** Advanced Imaging Research Center, Oregon Health & Science University, Portland, OR USA; Knight Cardiovascular Institute, Oregon Health & Science University, Portland, OR USA; Division of Cardiovascular Medicine, Oregon Health & Science University, Portland, OR USA

## Background

The *equilibrium* cellular water efflux rate constant [k_io_; mean water lifetime inverse] from contrast agent [CA]-enhanced MRI measures on-going cellular Na^+^,K^+^-ATPase activity [turnover]. Good literature [4 different labs] agreement shows substantial k_io_ decreases in myocardial ischemia, hypertension, or infarct regions (Table). The 3 methods used differ in extracellular ("outside") CA_o_ level manipulation to change the MR shutter-speed relative to k_io_ and the MR exchange condition reached: A) CA_o_ steady-state, slow-exchange-regime; B) CA_o_ titration, fast-exchange-regime [FXR]; and C) CA_o_ wash-out, FXR. The independent intracellular volume fraction [ICV] - cell density•volume product and ≈ 1 - ECV [extracellular volume fraction] - also decreases in pathology. We hypothesize that k_io_ mapping shows metabolic compromise most effectively. We report initial experience with tissue near a repaired ventricular septal defect [VSD].

## Methods

We acquired serial 1.5T ^1^H_2_O T_1_-weighted data from a 27 yo male before and 3 times after a bolus IV 0.15 mmol/kg CA [Omniscan] injection. Quantitative Look-Locker T_1_ measurements [non-selective inversion, 21 recovery times] imaged an 8 mm slice with a mid-ventricular short axis location inferior to the VSD patch. Method C (CA_o_ wash-out, FXR) determined k_io_ and ICV values in six LV wall segments.

## Results

The Figure shows a post-CA T_1_-w image: the endo- and epicardial LV wall edges as bright orange and green, respectively [light orange circle, an LV ROI]. Segmental ICV and k_io_ values are given (yellow). Segments S5 and S6 comprise the septum. The ICV values for segments S1 - S4 are reasonable for normal myocardium (Table). Thus, we have indicated (*) a control myocardial k_io_ value [5 s^-1^, Table], since the CA wash-out data quantity [3 points] and quality from these normal myocardium segments yielded insufficient precision. Interestingly, the k_io_ value is reduced [4.5 s^-1^] in segment S6, and dramatically so [1.7 s^-1^; 66%↓] in segment S5, immediately inferior to the VSD patch.

**Figure 1:**
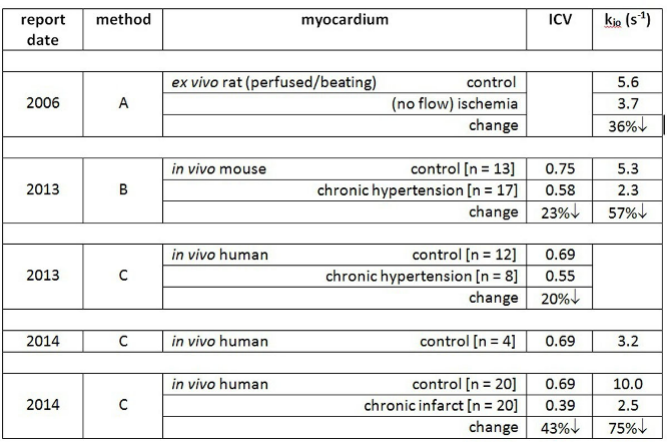
Literature reports of active trans-membrane water cycling [k_io_] and intracellular volume fraction [ICV] values in normal and pathological myocardia.

**Figure 2:**
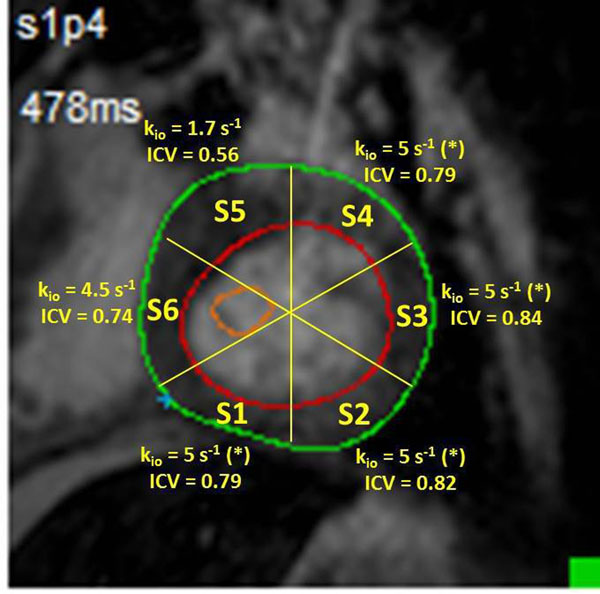
Short axis T_1_-w image slice inferior to VSD patch. The k_io_ and ICV values of six LV wall segments are given. k_io_ and ICV are reduced (66% and 30%, respectively) in segment S5,immediately below the patch.

## Conclusions

The k_io_ biomarker is a sensitive measure of on-going myocardial metabolic activity. Our result suggests that tissue nearby a VSD patch can be, or become, metabolically compromised.

The ultimate goal is pixel-wise k_io_ and ICV maps. [Here, nominal voxels are 2x2x8 mm^3^ = 32 μL.] For this, one needs data with good S/N and more than 3 wash-out points. Also, method C has systematic error absent in methods A and B, which cannot be used for humans. It assumes the CA_o_ concentration equals that of CA_p_ [in plasma] during wash-out. This is invalid for finite CA intravasation kinetics, which may be particularly slow in myocardial lesions due to common reduced vascularization. Possible k_io_ and ICV underestimations can be corrected using K^trans^ [the CA extravasation transfer constant] from the bolus tissue wash-in time-course to calculate the CA intravasation rate constant.

## Funding

NIH: RO1-NS040801.

